# Srxn1 Overexpression Protect Against Cardiac Remodelling by Inhibiting Oxidative Stress and Inflammation

**DOI:** 10.1111/jcmm.70432

**Published:** 2025-03-20

**Authors:** Huibo Wang, Ying Yang, Yong Ye, Xing Wei, Shen Chen, Bin Cheng, Yunbo Lv

**Affiliations:** ^1^ Department of Cardiology, the First College of Clinical Medical Science China Three Gorges University & Yichang Central People's Hospital; Institute of Cardiovascular Diseases, China Three Gorges University; Hu Bei Clinical Research Center for Ischemic Cardiovascular Disease Yichang Hubei China; ^2^ Department of Radiology, the First College of Clinical Medical Science China Three Gorges University & Yichang Central People's Hospital Yichang Hubei China

**Keywords:** heart failure, inflammation, oxidative stress, Srxn1

## Abstract

Oxidative stress and inflammation are common medical issues contributing to the onset and progression of heart failure (HF). Sulfiredoxin 1 (Srxn1) is a key regulatory factor in the antioxidant response. This study aimed to examine the effect of Srxn1 in HF. We utilised transcriptome sequencing to screen for differentially expressed genes in cardiac remodelling. We overexpressed Srxn1 in the hearts using an adeno‐associated virus 9 (AAV9) system through tail vein injection. C57BL/6 mice were subjected to transverse aortic constriction (TAC) for 4 weeks. Echocardiography was used to evaluate cardiac function, and cardiac remodelling was estimated by histopathology and molecular techniques. In addition, H9C2 cells were stimulated by Ang II to establish an in vitro model of cardiomyocyte hypertrophy, and the effects of Srxn1 overexpression on the inflammatory pathways and oxidative stress in Ang II‐stimulated H9C2 cells were examined. We found that Srxn1 is downregulated after cardiac remodelling by transcriptome sequencing. Our results revealed down‐regulated levels of Srxn1 in murine hearts subjected to TAC treatment, and H9C2 challenged with Ang II. Moreover, compared with WT mice, AAV‐9‐Srxn1 mice exhibited dramatically ameliorated TAC‐induced cardiac dysfunction, hypertrophy, fibrosis, oxidative stress, and inflammation. In terms of mechanism, both in vitro and in vivo experiments confirmed that the potential positive impacts may be linked to the inhibition of TLR4/NF‐κB signalling. In summary, this study is the first to demonstrate the protective effects of Srxn1 against TAC‐induced cardiac oxidative stress and inflammation, which are induced by the inhibited activation of the TLR4/NF‐κB signalling pathway.

## 1 Background

Heart failure (HF) severely affects many people around the world, with approximately 64 million individuals impacted. Furthermore, the prevalence of this disease is steadily increasing. The rising incidence of HF may be partly due to the aging population and high prevalence of cardiovascular risk factors such as hypertension, diabetes, and obesity, among which hypertension is the main risk factor for HF [1]. Although there have been significant advances in current treatment methods, the mortality rate has not declined [2]. Therefore, elucidating the pathogenesis and identifying effective therapeutic targets are crucial for reducing the mortality rate among patients with HF.

In recent years, oxidative stress and inflammation have attracted attention as key pathophysiological factors in HF [3]. Clinical and experimental studies have provided substantial evidence that oxidative stress (defined as a disturbance in the balance between reactive oxygen species (ROS) and antioxidants) is increased, leading to cardiac remodelling and HF [4, 5]. HF is characterised by a systemic pro‐inflammatory state, evidenced by high levels of circulating inflammatory mediators such as tumour necrosis factor‐α (TNF‐α) and interleukin (IL)‐6 [6]. Therefore, regulating oxidative stress and inflammation may provide a direction for the treatment of HF.

Sulfiredoxin 1 (Srxn1) is a key regulatory factor in the antioxidant response of eukaryotic cells [7, 8]. Some studies suggest that Srxn1 promotes survival and metastasis in various malignancies [8, 9, 10]. Previous research has found that Srxn1 enhances the survival of cardiac progenitor cells under oxidative stress by upregulating the extracellular regulated protein kinases (ERK)/ Nuclear factor erythroid 2‐related factor 2 (NRF2) signalling pathway [11, 12]. Srxn1 protects myocardial cells from ischemia/reperfusion injury by inhibiting the mitochondrial apoptosis pathway regulated by phosphatidylinositol‐3‐kinase (PI3K)/ protein kinase B (AKT) [13]. However, the role of Srxn1 in HF remains unclear. We utilised transcriptome sequencing to screen for differentially expressed genes involved in cardiac remodelling and, through validation, identified Srxn1 as a key gene in cardiac reconstruction. This study aims to explore the role and underlying mechanisms of Srxn1 in the development and progression of HF.

### 1.1 Animals and Treatment

Male C57BL/6 mice (8‐ to 10‐week‐old) were purchased from the Chinese Academy of Medical Sciences and performed in accordance with the Guidelines for the Care and Use of Laboratory Animals published by the United States National Institutes of Health (NIH Publication, revised 2011). The use of animals was reviewed and approved by the Ethics Committee of the China Three Gorges University. Mice were allowed 1 week to acclimatise to a stable environment before experiments began. Mice were individually housed in plastic cages with bedding, ad libitum food, and tap water. The cages were maintained at 22°C ± 2°C and a 12:12‐h light/dark cycle. C57BL/6 mice were randomly divided into four groups: sham + AAV9‐GFP, sham + AAV9‐Srxn1, Thoracic aortic constriction (TAC) + AAV9‐GFP, and TAC + AAV9‐Srxn1. To overexpress Srxn1 in mice, the mice were intravenously injected via the tail vein with adeno‐associated viral 9 vector (AAV9) (Genechem Co. Ltd.; Shanghai, China) carrying Srxn1 (AAV9‐Srxn1) or AAV9 vector that expressed green fluorescent protein (GFP) as control (AAV9‐GFP). AAV9‐Srxn1 or AAV9‐GFP were intramyocardially injected with 2 × 10^10^ viral genome particles per mouse. Following 1 week of AAV9‐Srxn1 or AAV9‐GFP injection, mice were subjected to perform transverse aortic constriction (TAC)‐induced HF model. The TAC and sham surgeries were performed as described previously [14]. All the mice were monitored and weighed weekly and were sacrificed following a 4‐week TAC‐induced HF mouse model, with hearts collected for further experiment.

### 1.2 Echocardiography and Hemodynamics

Echocardiography analysis was performed as previously reported [15]. In brief, echocardiography was performed under continuous anaesthesia with 1.5%–2% isoflurane, using a Mylab30CV (ESAOTE) ultrasound system with a 15Mz probe. Cardiac measurements included examination of left ventricular end‐diastolic diameter (LVEDD), left ventricular end‐systolic diameter (LVESD), left ventricular ejection fraction (LVEF) and left ventricular fractional shortening (LVFS).

### 1.3 Histological Analysis

Embedded heart tissues were cut into 5 μm sections. Masson staining was used to analyse the extent of myocardial fibrosis, and wheat germ agglutinin (WGA, L4895, Sigma Aldrich, USA) to assess cardiac hypertrophy, according to the manufacturers' instructions. Cardiac morphology and the extent of cardiac fibrosis and hypertrophy were assessed using ImageJ software (NIH Image, Bethesda, MD, USA).

### 1.4 Immunohistochemistry (IHC)

The mouse heart tissue sections were subjected to antigen retrieval and subsequently washed in 3% H_2_O_2_ at room temperature for 20 min. Following this, the sections were blocked with 10% bovine serum albumin (BSA) at 37°C for 30 min. The Srxn1 primary antibody (1:50, Proteintech, Illinois, IL, USA) was then applied, and the sections were incubated at 4°C overnight. Subsequently, the sections were washed with PBS and treated with the Goat anti‐Rabbit Detection Kit (BLRE006‐200 T, Biolight) at 37°C for 1 h. Positive signals on the slices were visualised using diaminobenzidine working solution (ZLI‐9018, ZSGB‐BIO) after washing with PBS. Haematoxylin staining (G1004, Servicebio) was performed, followed by washing with ddH_2_O. Finally, the slices were sealed with a resin sealant (BA‐7004, Baso), and images were obtained using Digital Pathology Slide Scanners (Aperio Versa 200, Leica).

### 1.5 Immunofluorescence Staining

Immunofluorescence was also performed on paraffin sections using the particular macrophage antibody F4/80 (#AG4753, NeoBioscience Technology Co., China) to stain the inflammatory cells. An OLYMPUS DX51 fluorescent microscope (Tokyo, Japan) was used to take the photos. Each heart sample's stained sections were examined and captured on camera using a microscopy (×200) with 20 randomly selected fields of vision. Each sample's infiltrating macrophage count was determined.

### 1.6 ROS Detection

To detect ROS, DHE staining was conducted. Briefly, cryosections of fresh heart samples were stained with DHE (10 μM, #S0063, Beyotime, China) for 30 min at 37°C to detect ROS production. Pictures were taken with an OLYMPUS DX51 fluorescence microscope (Tokyo, Japan) and analysed with Image J software. The stained sections were observed and photographed under microscopy (×200) of 20 random fields of view of each heart sample. Furthermore, fresh heart samples were homogenised on ice and centrifuged at 3000 rpm for 15 min. After that, the supernatants were collected to measure the content of malondialdehyde (MDA, #S0131S, NeoBioscience Technology Co., China), 4‐HNE(#H268‐1‐2, Nanjing Jiancheng, China), total superoxide Dismutase (SOD, #S0086, NeoBioscience Technology Co., China) activity, and catalase (CAT, #S0082, NeoBioscience Technology Co., China) activity by commercial kits according to manufacturer's instructions [15].

### 1.7 Cell Culture

H9C2 cells were obtained from the Cell Bank of the Chinese Academy of Sciences (Shanghai, China) and cultured in Dulbecco's modified Eagle's medium with fetal bovine serum (10%), streptomycin (1%), and penicillin. The culture conditions contained a humidified atmosphere (95% air and 5% CO_2_ at 37°C). The H9C2 cells were incubated with Ang (1 μM) for 1 h [16]. To overexpress Srxn1, cells were transfected with adenoviral vectors (Genechem Co. Ltd.; Shanghai, China) carrying Srxn1 (Ad‐Srxn1) or adenoviral null vectors (Ad‐null) for 24 h; then the H9C2 cells were infected with lipopolysaccharide (LPS, #ST1470, NeoBioscience Technology Co., China, 100 ng/mL) or PBS for 24 h to further verify the role of TLR4/NF‐κB signalling in the protective role of Srxn1 overexpression.

### 1.8 RT‐qPCR

Total RNA was extracted from cardiac tissues or cells using Trizol reagent (Invitrogen). First‐strand cDNA was synthesised from total RNA using Prime Script RT Master Mix (Takara, Tokyo, Japan). RT‐qPCR was performed in a 25 μL reaction on the CFX96 Real‐Time PCR Detection System (Bio‐Rad Laboratories), including 0.4 μmol/L primers, 50 ng of cDNA, and 12.5 μL TB Green Premix Ex Taq II (Takara). The expression levels of target genes were normalised to the expression levels of beta‐actin, which was considered an endogenous internal control. The primer sequences were as exhibited in Table 1.

**TABLE 1:** Mouse primers for RT‐PCR.

Gene	Forward primers	Reverse primers
ANP	GGAGCAAATCCCGTATACAGTG	CTCTGAGACGGGTTGACTTCC
BNP	TCAAAGGACCAAGGCCCTAC	CTAAAACAACCTCAGCCCGTC
Collagen‐I	GTCCCTGAAGTCAGCTGCATA	TGGGACAGTCCAGTTCTTCAT
CTGF	CAAAGCAGCTGCAAATACCA	GGCCAAATGTGTCTTCCAGT
IL‐1β	TCAAGCAGAGCACAGACCTG	GAAGACACGGGTTCCATGGT
IL‐6	GCAAGAGACTTCCAGCCAGT	CTGGTCTGTTGTGGGTGGTA
TNF‐α	GCCCAGACCCTCACACTC	CCACTCCAGCTGCTCCTCT
GAPDH	CGCTAACATCAAATGGGGTG	TTGCTGACAATCTTGAGGGAG

### 1.9 Western Blotting

Western blotting was performed to evaluate protein expression levels as described previously [17, 18]. This manuscript's primary antibodies are: Srxn1 (1:1000, #ab203613, Abcam), TLR4 (1:1000, #19811‐1‐AP, Proteintech Group), p‐p65 (1:1000, #3033, CST), p65 (1:1000, #8242, CST) and GAPDH (1:10000, #ab181602, Abcam). Incubate the blots with a 1:1000 diluted secondary antibody against rabbit immunoglobulin G (1:1000, #ab205718, Abcam) at room temperature for 1 h, wash, and visualise. The total protein levels were normalised to GAPDH.

### 1.10 Transcriptome Sequencing

Myocardial tissues were collected from mice in both the sham‐operated group(Sham) and the transverse aortic constriction (TAC) group. Total RNA was extracted and reverse‐transcribed into cDNA. Subsequently, sequencing was performed using a second‐generation high‐throughput sequencing platform. The sequencing results were analysed using R language, and volcano plots and heatmaps were generated to screen for differentially expressed genes (DEGs, *the definition of a differentially expressed gene is one with a Log*_*2*_ *FC less than − 1 or greater than 1, and an adjusted P‐value less than 0.05*). Protein–protein interaction analysis of the DEGs was conducted using STRING to identify functionally significant genes.

### 1.11 Statistical Analysis

All data are expressed as mean ± SEM. The normality of data distribution was initially assessed using the Shapiro–Wilk normality test. To ascertain statistical disparities, a two‐tailed unpaired Student's t‐test was employed for comparisons between two distinct groups. One‐way analysis of variance (ANOVA) was conducted to evaluate the differences between the groups in GraphPad Prism software. Differences were considered statistically significant at *p* < 0.05.

## 2 Results

### 2.1 Transcriptome Sequencing to Detect DEGs in Cardiac Remodelling

**FIGURE 1:**
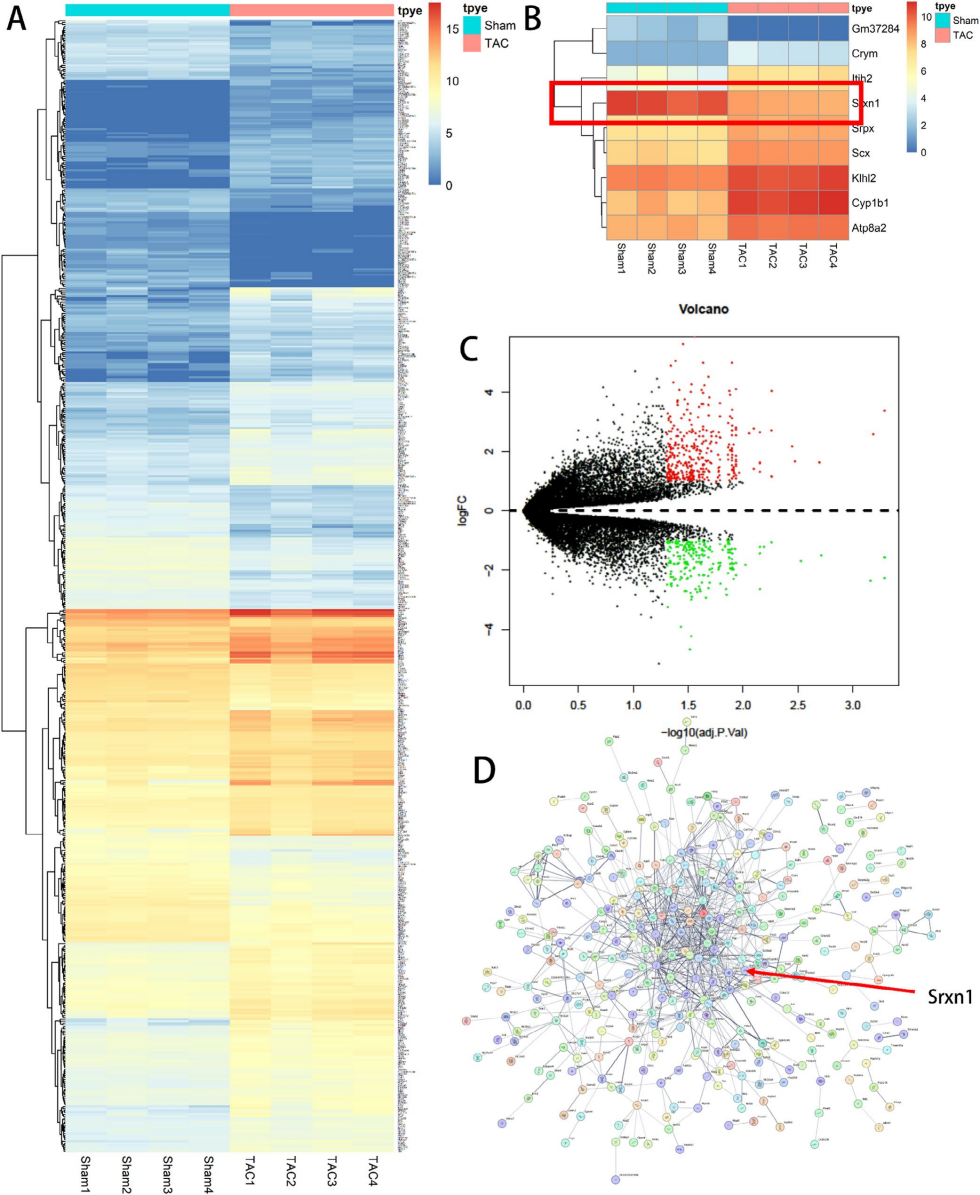
Identification of Differentially Expressed Genes During Cardiac Remodelling through Transcriptome Sequencing. (A) Differentially expressed genes (with Log_2_ FC > 1 or < −1, *and an adjusted P‐value less than 0.05*) were identified using R language, and a heatmap of all differentially expressed genes was generated, with different colours representing the expression levels of these genes across samples. (B) A heatmap of a subset of differentially expressed genes. (C) A volcano plot of all differentially expressed genes was created using R language, with red dots indicating upregulated genes (Log_2_ FC > 1) and green dots indicating downregulated genes (Log_2_ FC < −1), *and an adjusted P‐value less than 0.05*. (D) Protein–protein interactions among the differentially expressed genes were analysed using STRING; FC = fold change.

To detect differentially expressed genes during cardiac remodelling, we performed transcriptome sequencing on myocardial tissues from four Sham groups and four TAC groups. We identified 584 differentially expressed genes during cardiac remodelling, with 230 downregulated genes and 354 upregulated genes. Using R language, we generated heatmaps (Figure 1A,B) and volcano plots (Figure 1C) to visualise the differentially expressed genes (*the definition of a differentially expressed gene is one with a Log*_*2*_ *FC less than − 1 or greater than 1, and an adjusted P‐value less than 0.05*). Additionally, we conducted protein–protein interaction analysis of the differentially expressed genes using the STRING website and, combined with relevant literature, initially identified SRXN1 as our gene of interest for further study.

### 2.2 Srxn1 Was Decreased Under HF Condition

**FIGURE 2:**
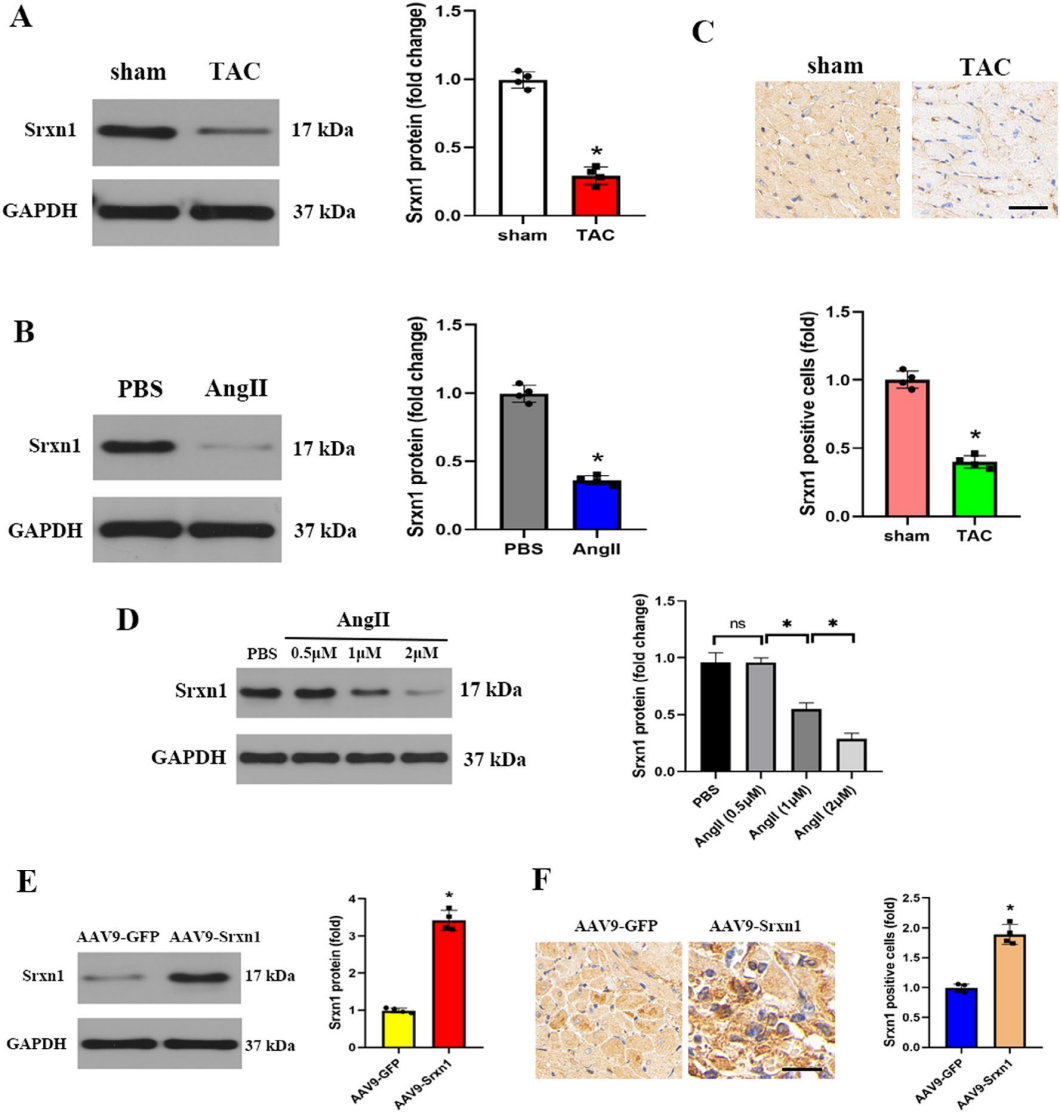
Srxn1 was decreased under HF condition. (A) Representative western blot and quantitative data of Srxn1 protein expression in mice (*n* = 4 per group). (B) Representative western blot and quantitative data of Srxn1 protein expression in vitro (*n* = 4 per group). (C) Representative IHC staining and quantitative data of Srxn1 positive cells in mice (*n* = 4 per group). (D) Srxn1 expression in H9C2 cells. H9C2 cells were subjected to treatment with different concentrations of Ang II and then collected for western blot detection. (E) Representative western blot and quantitative data of Srxn1 protein expression in AAV9‐GFP and AAV9‐Srxn1 mice (*n* = 4 per group). (F) Representative IHC staining and quantitative data of Srxn1 positive cells in AAV9‐GFP and AAV9‐Srxn1 mice (*n* = 4 per group). *p < 0.05. IHC = immunohistochemistry.

To clarify the relationship between Srxn1 and HF, we first detected the protein expression level of Srxn1 in TAC‐induced hearts. Srxn1 protein expression was significantly downregulated in TAC hearts (Figure 2A). We then checked the protein expression of Srxn1 in H9C2 cells; consistent with the in vivo result, the protein expression of Srxn1 was decreased following Ang II treatment in vitro (Figure 2B). IHC staining further indicated a significantly decreased number of Srxn1 positive cells in TAC‐treated ventricles compared to sham ventricles (Figure 2C). In vitro studies used different doses of Ang II to check the optimal dosage of Ang II, and the result demonstrated that Ang II at the dosage of 1 μM significantly decreased Srxn1 protein expression, and this dosage (1 μM) was used in the further in vitro experiment (Figure 2D). Then AAV9 was injected via the tail vein to verify the role of Srxn1 in vivo. Western blot analysis revealed that Srxn1 overexpression by AAV9‐Srxn1 notably increased Srxn1 protein expression compared to the AAV9‐GFP group (Figure 2E). IHC staining also verified that Srxn1 overexpression by AAV9‐Srxn1 significantly elevated Srxn1 expression compared to the AAV9‐GFP group (Figure 1F).

### 2.3 Srxn1 Overexpression Improved TAC‐Induced Cardiac Dysfunction

**FIGURE 3:**
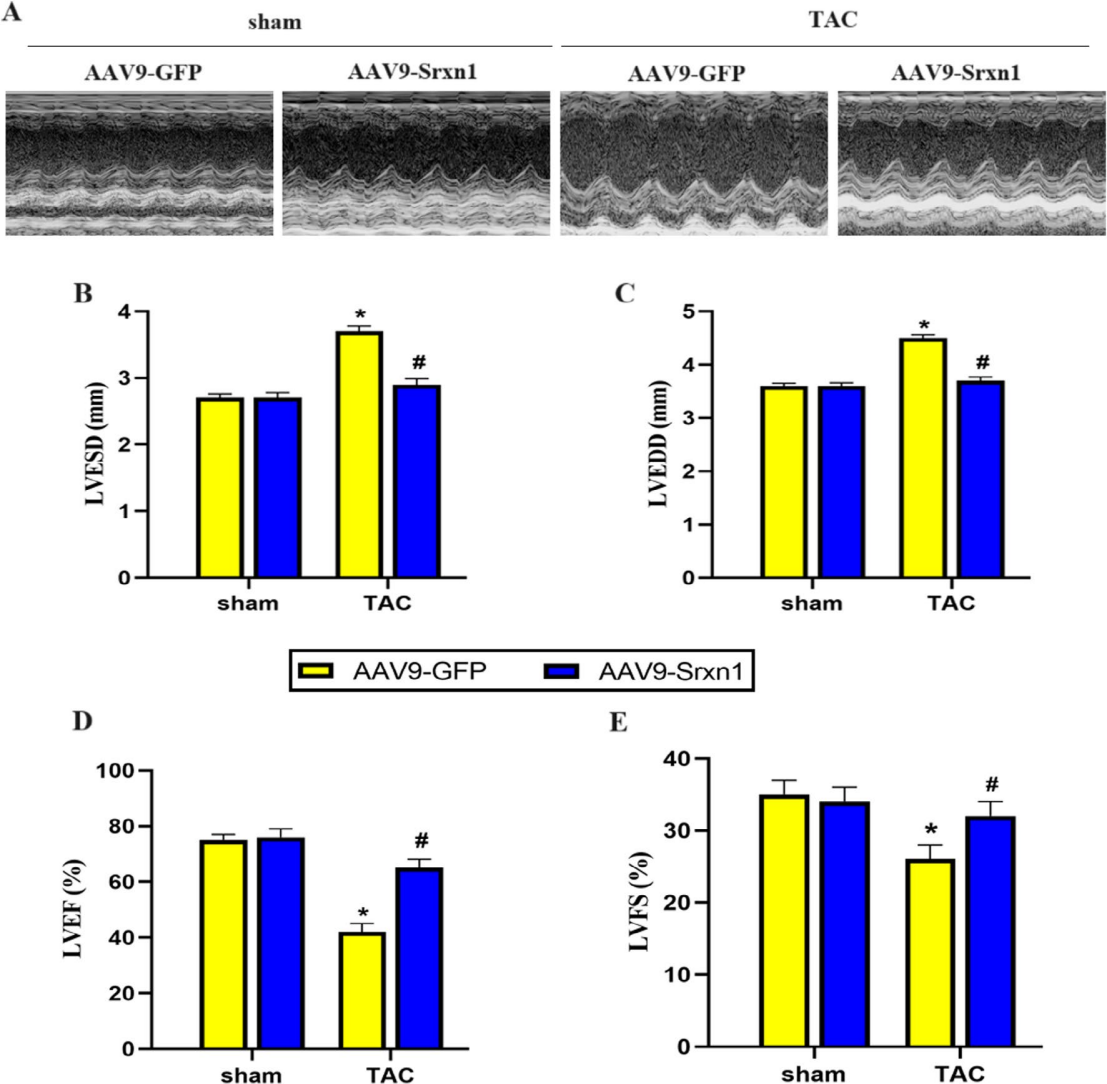
Srxn1 overexpression improved TAC‐induced cardiac dysfunction. (A) Representative echocardiograph image. (B, C) Statistical results of LVESD and LVEDD (*n* = 6 per group). (D, E) Statistical results of LVEF and LVFS (*n* = 6 per group). *p < 0.05 vs. sham+AAV9‐GFP group. #*p* < 0.05 vs. TAC + AAV9‐GFP group. LVEDD = Left Ventricular End Diastolic Diameter; LVESD = Left Ventricular End Systolic Diameter.

Then, we checked the effect of Srxn1 overexpression on TAC‐induced cardiac dysfunction. 4‐weeks after TAC, mice depicted cardiac enlargement and cardiac dysfunction, which manifested in increased LVESD, LVEDD, and decreased LVEF, LVFS compared to sham mice (Figure 3A–E). Furthermore, Srxn1 overexpression significantly reduced LVESD, LVEDD, and increased LVEF, LVFS compared to sham mice, indicating that Srxn1 overexpression could ameliorate TAC‐induced cardiac dysfunction (Figure 3A–E). These data implicate that Srxn1 overexpression improved TAC‐induced cardiac dysfunction.

### 2.4 Srxn1 Overexpression Alleviated TAC‐Induced Cardiac Hypertrophy and Fibrosis

**FIGURE 4:**
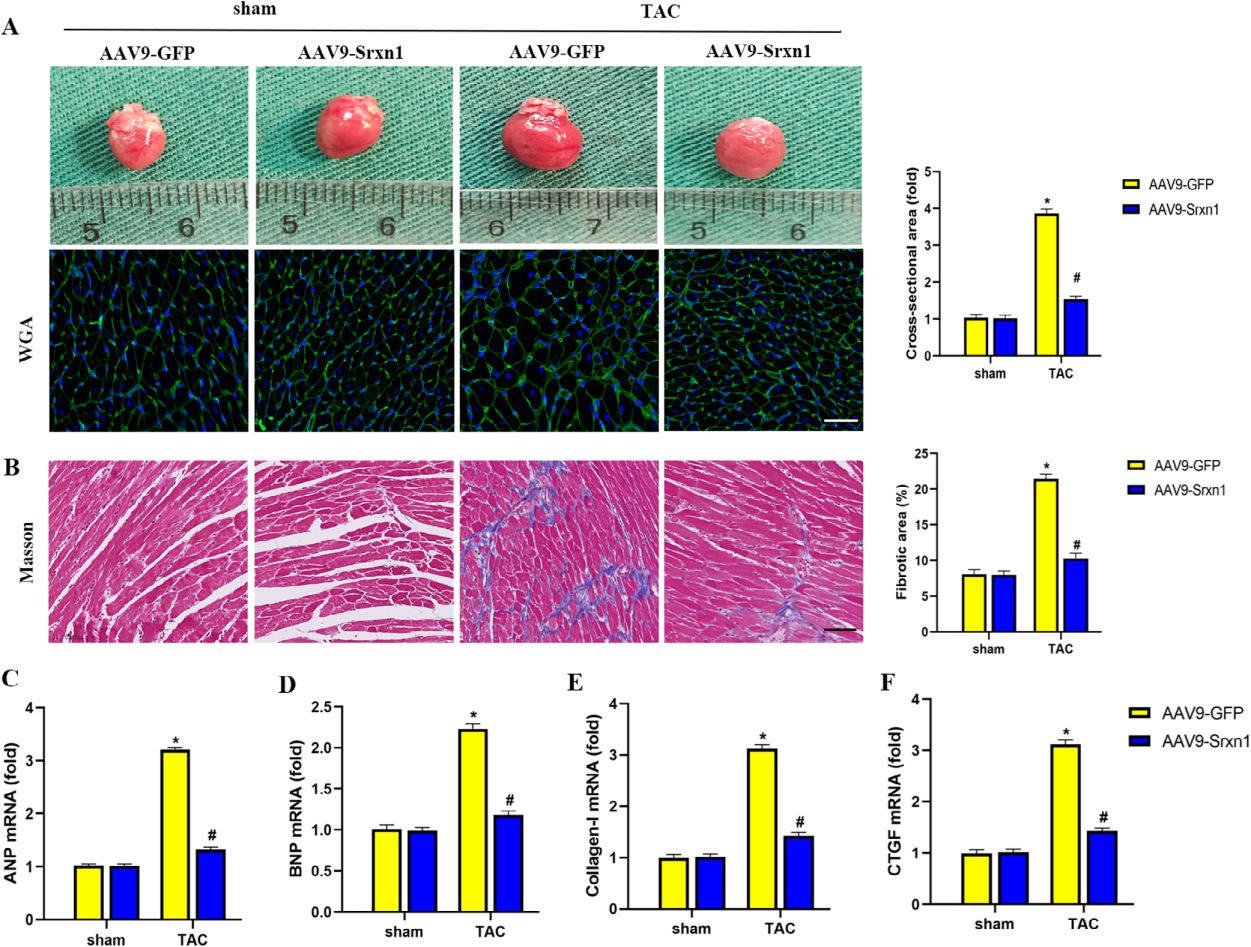
Srxn1 overexpression alleviated TAC‐induced cardiac hypertrophy and fibrosis. (A) Representative images of gross heart, WGA‐stained heart sections and quantitative results of cross‐sectional area (*n* = 5 per group). (B) Representative images of masson‐stained heart sections and quantitative results of fibrotic area (*n* = 5 per group). (C–D) Quantitative results of mRNA levels of ANP and BNP (*n* = 4 per group). (E, F) Quantitative results of mRNA levels of collagen‐I and CTGF (*n* = 4 per group). *p < 0.05 vs. sham+AAV9‐GFP group. #*p* < 0.05 vs. TAC + AAV9‐GFP group. ANP = atrial natriuretic peptide; BNP = Brain natriuretic peptide; CTGF = connective tissue growth factor; TAC = Thoracic aortic constriction; WGA = Wheat germ agglutinin.

Cardiac hypertrophy and cardiac fibrosis are the most characteristic features in TAC‐induced HF. However, whether Srxn1 overexpression has an effect on TAC‐induced cardiac hypertrophy and cardiac fibrosis has not been clarified. WGA staining revealed that compared to the sham group, significantly increased cross‐sectional areas were found in TAC hearts (Figure 4A). The result was further verified by RT‐PCR, demonstrating significantly increased ANP and BNP mRNA in TAC hearts compared to sham hearts (Figure 4C,D), indicating cardiac hypertrophy in TAC hearts. Moreover, Masson staining indicated that compared to the sham group, significantly increased cardiac fibrosis was found in TAC hearts (Figure 4B), and the result was further verified by RT‐PCR results showing significantly increased collagen‐I and CTGF mRNA levels in TAC hearts compared to sham hearts (Figure 4E,F). Unexpectedly, all these changes induced by TAC in cardiac hypertrophy and cardiac fibrosis were significantly reversed by Srxn1 overexpression. These data revealed that Srxn1 overexpression protects against TAC‐induced cardiac hypertrophy and cardiac fibrosis.

### 2.5 Srxn1 Overexpression Attenuates TAC‐Induced Oxidative Stress

**FIGURE 5:**
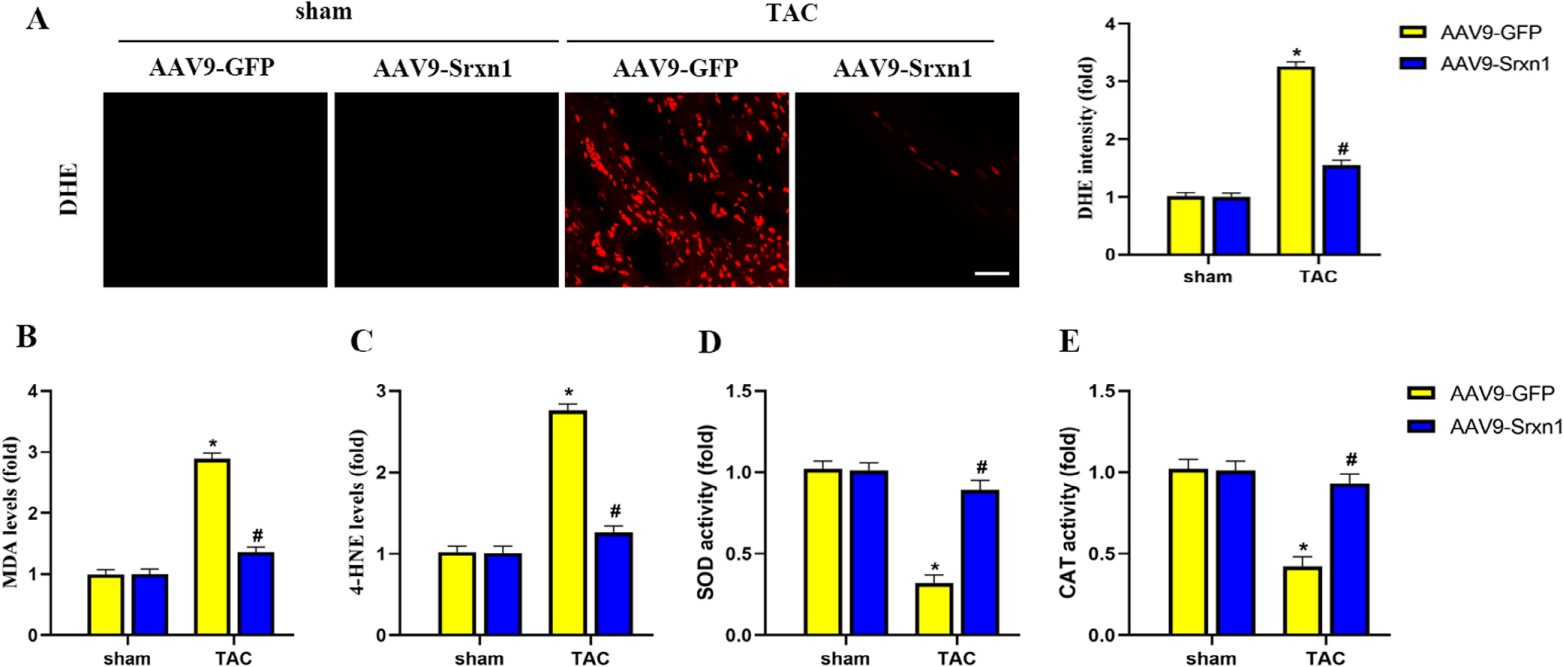
Srxn1 overexpression attenuated TAC‐induced oxidative stress. (A) Representative images and quantitative results of DHE stained heart sections (*n* = 5 per group). (B–E) Srxn1 overexpression treatment significantly decreased MDA levels, 4‐HNE levels, increased SOD activity and CAT activity in heart tissues (*n* = 5 per group). *p < 0.05 vs. sham+AAV9‐GFP group. #*p* < 0.05 vs. TAC + AAV9‐GFP group. 4‐HNE =4‐Hydroxynonenal; CAT = catalase; DHE = dihydroethidium; MDA = malondialdehyde; SOD = superoxide dismutase.

Oxidative stress plays an important role in TAC‐induced HF. we then tested the effect of Srxn1 overexpression on TAC‐induced oxidative stress. DHE staining showed a significant increase in cardiac ROS in the TAC group compared to the sham group, while compared to the TAC group, cardiac ROS significantly decreased after Srxn1 overexpression (Figure 5A). By using biochemical testing, the antioxidant benefits of Srxn1 overexpression were further verified. Figure 5B–E depicted that Srxn1 overexpression treatment significantly decreased MDA levels and 4‐HNE levels, and increased SOD activity and CAT activity. These results revealed that Srxn1 overexpression could reverse TAC‐induced oxidative stress.

### 2.6 Srxn1 Overexpression Inhibited TAC‐Induced Inflammation

**FIGURE 6:**
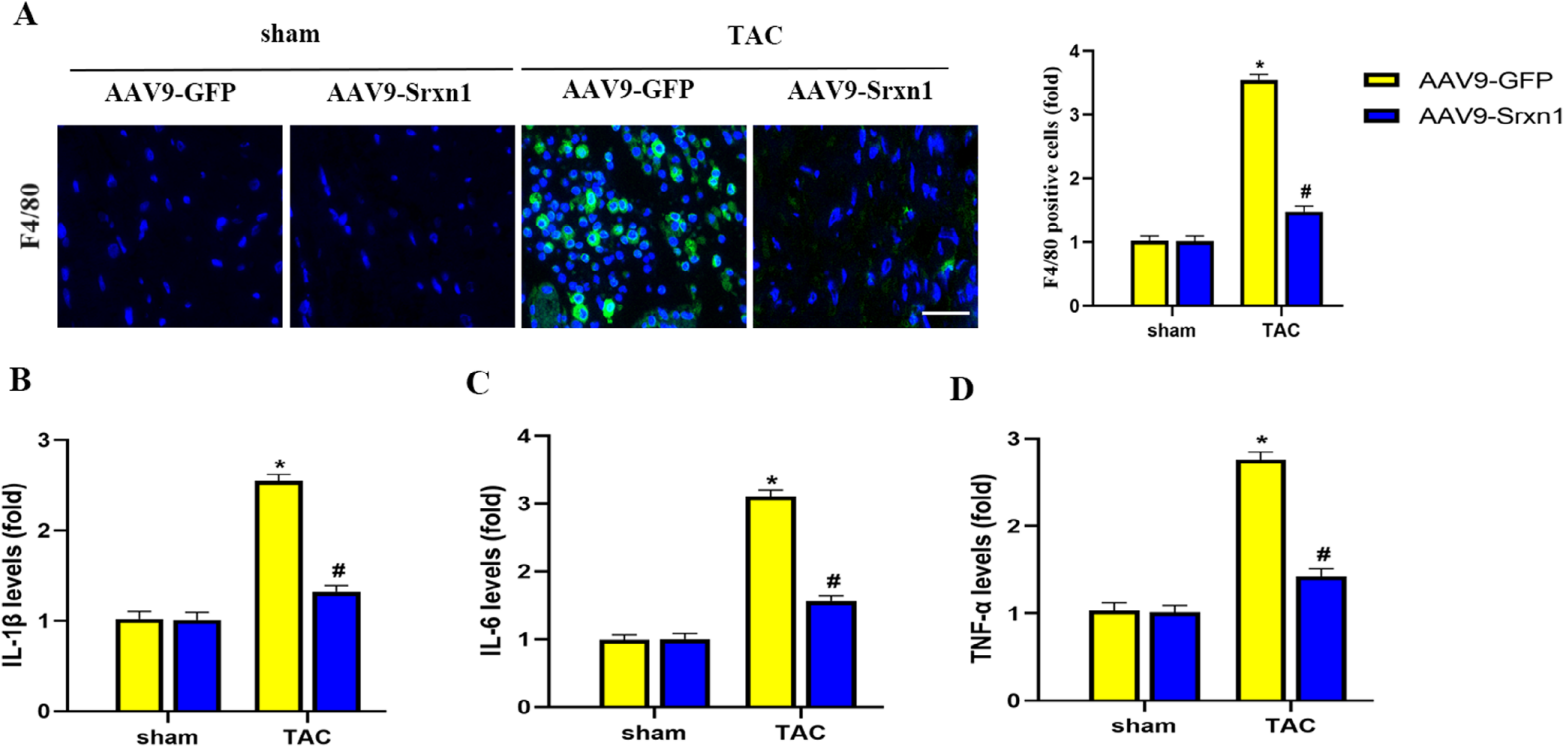
Srxn1 overexpression inhibited TAC‐induced inflammation. (A) Representative images and quantitative results of F4/80‐stained heart sections (*n* = 5 per group). (B–D) Quantitative results of the serum levels of IL‐1β, IL‐6 and TNF‐α (*n* = 5 per group). *p < 0.05 vs. sham+AAV9‐GFP group. #*p* < 0.05 vs. TAC + AAV9‐GFP group.

Cardiac inflammatory response is a hallmark in TAC‐induced HF. F4/80 immunofluorescence staining suggested enhanced macrophage infiltration in the TAC hearts, as exhibited in Figure 6A. Srxn1 overexpression significantly decreased the infiltration of macrophages induced by TAC. In addition, compared to the sham group, the plasma levels of IL‐1β, IL‐6, and TNF‐α were markedly elevated in TAC mice. Srxn1 overexpression significantly decreased the production of pro‐inflammatory cytokines induced by TAC (Figure 6B–D). These findings demonstrated that Srxn1 overexpression significantly inhibited TAC‐induced cardiac inflammatory response.

### 2.7 TLR4/NF‐κB Signalling Contributes to the Cardioprotective Effect of Srxn1 Overexpression

**FIGURE 7:**
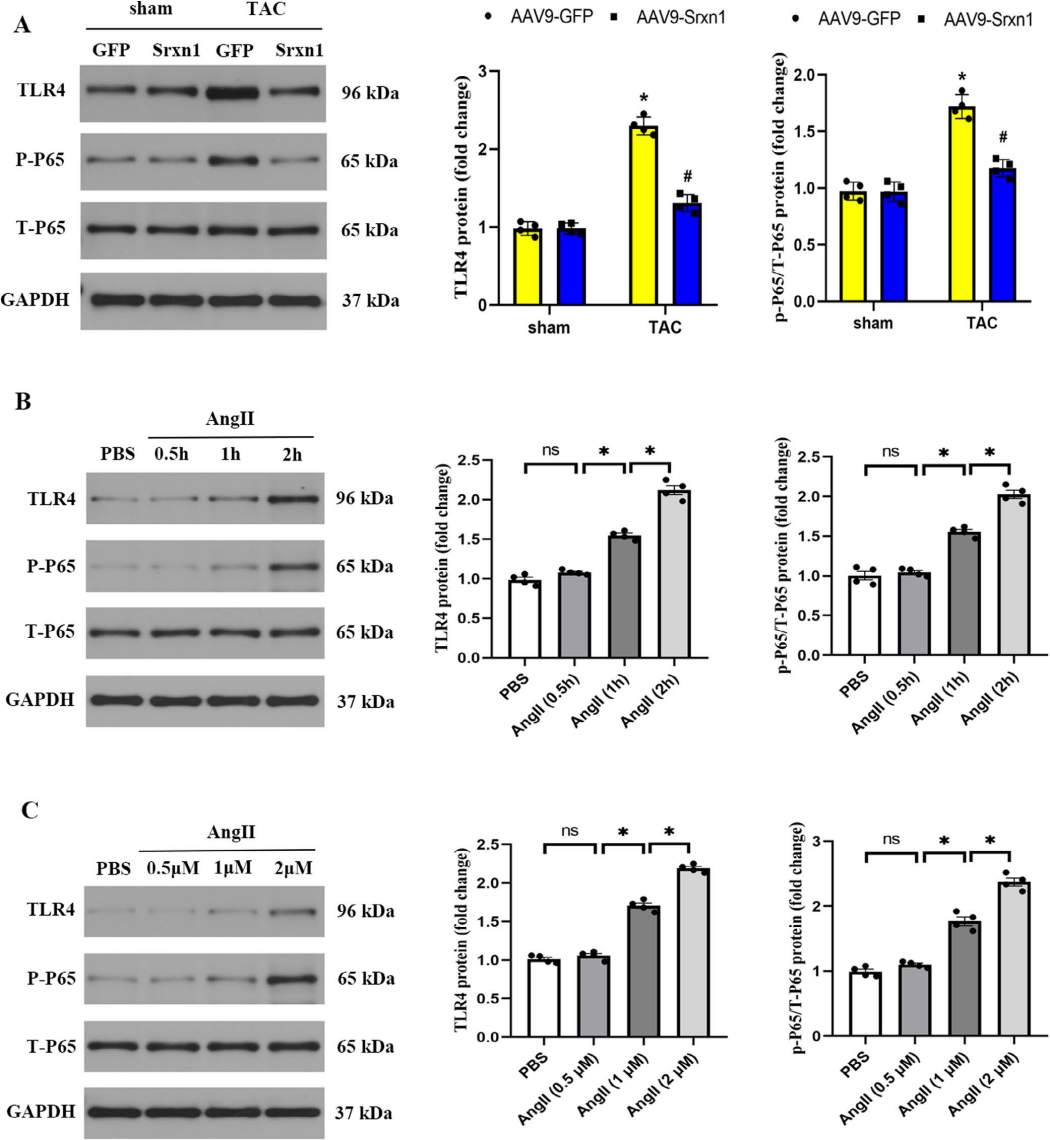
TLR4/NF‐κB signalling contributes to the cardioprotective effect Srxn1 overexpression. (A) Representative western blot and quantitative data of TLR4, p‐P65 and T‐P65 protein expression (*n* = 4 per group). *p < 0.05 vs. sham+AAV9‐GFP group. #*p* < 0.05 vs. TAC + AAV9‐GFP group. (B) Srxn1 expression in H9C2 cells. H9C2 cells were subjected to treatment with Ang II at different time points and then collected for western blot detection. *p < 0.05. (C) Srxn1 expression in H9C2 cells. H9C2 cells were subjected to treatment with Ang II at different dosage and then collected for western blot detection. *p < 0.05.

TLR4/NF‐κB signalling pathway plays an important role in oxidative stress and inflammation. However, the role of Srxn1 overexpression in the TLR4/NF‐κB signalling pathway induced by TAC has not been explored. We then checked the TLR4/NF‐κB signalling pathway via western blot. Figure 7 indicated that the activated TLR4/NF‐κB signalling pathway was found in TAC hearts compared to sham hearts, as reflected by increased TLR4 protein expression and p‐P65 protein expression. Srxn1 overexpression significantly inhibited the TLR4/NF‐κB signalling pathway by decreasing TLR4 protein expression and p‐P65 protein expression. In vitro studies showed that TLR4 and P‐P65/T‐P65 protein expression were significantly upregulated in H9C2 cells treated with Ang II (1 μM) for 1 h and 2 h in a time‐dependent manner (Figure 7B). Moreover, TLR4 and P‐P65/T‐P65 protein expression were significantly upregulated in H9C2 cells treated with Ang II for 1 μM and 2 μM in a dosage‐dependent manner (Figure 7C).

### 2.8 TLR4 Activation Offsets the Protective Role of Srxn1 Overexpression on Ang II‐Induced Oxidative Stress and Inflammation

**FIGURE 8:**
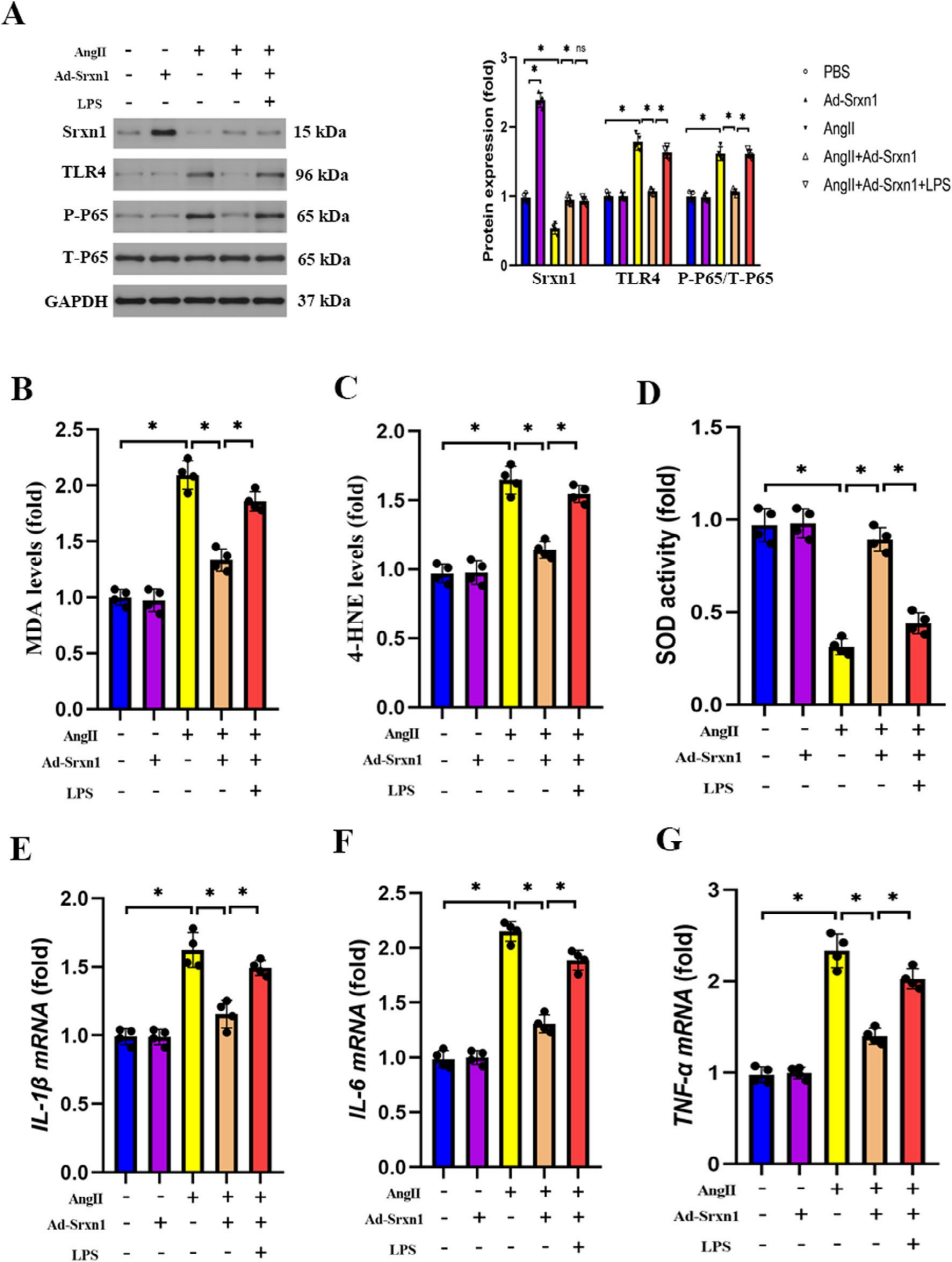
TLR4 activation offset the protective role of Srxn1 overexpression on Ang II‐induced oxidative stress and inflammation. (A) Representative western blot and quantitative data of Srxn1, TLR4, P‐P65 and T‐P65 protein expression in H9C2 cells (*n* = 4 per group). *p < 0.05. (B–D) Quantitative results of MDA, 4‐HNE levels and SOD activity in H9C2 cells in different groups (*n* = 4 per group). (E–G) Quantitative analysis of mRNA levels of IL‐1β, IL‐6 and TNF‐α (*n* = 4 per group). *p < 0.05.

To further verify the role of TLR4/NF‐κB signalling in the protective role of Srxn1 overexpression, H9C2 cells were treated with LPS, a specific agonist of TLR4. Similar to the results in vivo, Ang II significantly decreased Srxn1 levels and increased TLR4, P‐P65/TP65 levels compared to the control group. Srxn1 overexpression markedly increased Srxn1 levels and decreased TLR4, P‐P65/TP65 levels compared to the Ang II group (Figure 8A). However, LPS treatment significantly increased TLR4, P‐P65/TP65 levels compared to the Ang II + Ad‐Srxn1 group (Figure 8A). Similar to the results in vivo, Ang II significantly induced oxidative stress, which was reflected by increased MDA levels, 4‐HNE levels and decreased SOD activity levels compared to the control group. Srxn1 overexpression markedly reduced MDA levels, 4‐HNE levels and increased SOD activity levels (Figure 8B–D). However, LPS treatment significantly increased MDA levels, 4‐HNE levels and decreased SOD activity compared to the Ang II + Ad‐Srxn1 group, which offset the antioxidant effect of Srxn1 overexpression (Figure 8B–D). Moreover, consistent with the in vivo results, Ang II significantly increased the inflammatory response, which was reflected by increased *IL‐1β, IL‐6* and *TNF‐α* mRNA levels compared to the control group. Srxn1 overexpression markedly reduced *IL‐1β, IL‐6* and *TNF‐α* mRNA levels (Figure 8E–G). However, LPS treatment markedly increased *IL‐1β, IL‐6* and *TNF‐α* mRNA levels compared to the Ang II + Ad‐Srxn1 group, which offset the anti‐inflammatory effect of Srxn1 overexpression (Figure 8E–G). These results further verified the role of TLR4/NF‐κB signalling in the protective effect of Srxn1 overexpression on Ang II‐induced oxidative stress and inflammation.

## 3 Discussion

This study primarily investigates the mechanism of action of Srxn1 in HF. Our research offers a new direction for the treatment of HF. The study found that Srxn1 expression is significantly downregulated in myocardial tissue of HF models. Overexpression of Srxn1 can alleviate the deterioration of cardiac function in mice with HF. Overexpression of Srxn1 can inhibit oxidative stress and inflammation in myocardial tissue of mice with TAC‐induced HF. Furthermore, we have confirmed that overexpression of Srxn1 inhibits the activation of the TLR4/NF‐κB signalling pathway. Therefore, we believe that Srxn1 improves myocardial oxidative stress and inflammation in mice with HF by targeting the TLR4/NF‐κB signalling pathway.

Srxn1 plays a significant role in the development of many diseases. For example, Srxn1 expression was upregulated in mild acute pancreatitis but decreased in severe cases [19]. Srxn1 was found to be upregulated in samples of hepatocellular carcinoma [20]. Quantitative PCR and western blotting confirmed increased expression of Srxn1 and KRT6A mRNA and protein in lung cancer cell lines and non‐small cell lung cancer tissues [21]. Our research revealed a downregulation of Srxn1 expression in myocardial tissue of HF models, indicating that Srxn1 is involved in the regulation of HF onset. We found that upregulating Srxn1 could improve cardiac function, as well as reduce myocardial hypertrophy and fibrosis following HF. Pathological cardiac remodelling is a fundamental basis of HF [22]. Therefore, we have reason to believe that Srxn1 is an important target in the pathological remodelling following HF. Moreover, recent studies have found that upregulating Srxn1 can protect H9C2 cells from ischemia–reperfusion (I/R) injury, thus improving cardiac function [13]. Consequently, it is evident that Srxn1 is involved in protecting against cardiac dysfunction following HF.

Oxidative stress and inflammation have been shown to be key processes in pathological myocardial remodelling following HF [3, 23]. Inhibiting oxidative stress and inflammation can improve left ventricular remodelling and cardiac function in mice subjected to pressure overload‐induced HF [23, 24]. Furthermore, previous studies have found that inhibiting Srxn1 increased ROS and inflammatory responses in mice with acute pancreatitis, while overexpression of Srxn1 in acinar cells produced the opposite effect [19]. Upregulating Srxn1 significantly reduces cell apoptosis, ROS production, and pro‐inflammatory cytokine release in retinal ganglion cells induced by high glucose [25]. Knocking down Srxn1 leads to increased expression of pro‐inflammatory cytokines and chemokines such as TNF‐α, Myeloperoxidase, IL‐1β, and IL‐6 in astrocytes, thereby reducing their activity [26]. Consistent with our findings, overexpression of Srxn1 also inhibits oxidative stress and inflammatory responses in myocardial tissue of mice with TAC‐induced HF. Therefore, we believe that Srxn1 can influence the onset and progression of HF by regulating oxidative stress and inflammation during post‐HF pathological myocardial remodelling.

It has been well documented that TLR4/NF‐κB plays a pivotal role in oxidative stress and inflammation [27, 28]. Additionally, the TLR4/NF‐κB signalling pathway plays a significant role in pathological myocardial remodelling following HF [29]. To explore the potential mechanism by which Srxn1 ameliorates pathological myocardial remodelling after HF, this study examined the effects of Srxn1 on the TLR4/NF‐κB signalling pathway. We found that in both HF model mice and Ang II‐stimulated primary mouse cardiomyocytes, the expression of TLR4 and p‐p65 was significantly upregulated. Overexpression of Srxn1 reversed these changes. Furthermore, we discovered that TLR4 activation offset the beneficial effect of Srxn1 overexpression on Ang II‐induced oxidative stress and inflammation. Therefore, this study posits that Srxn1 exerts anti‐inflammatory and antioxidant effects by targeting the TLR4/NF‐κB signalling pathway, thereby improving pathological myocardial remodelling after HF.

## 4 Limitation

Clearly, there are several unresolved issues that merit further investigation. Firstly, while our data suggest that overexpression of Srxn1 may inhibit HF‐induced oxidative stress and inflammation, additional studies are necessary to elucidate the specific role of Srxn1 in cardiac oxidative stress and inflammation. Secondly, since our research utilised Srxn1 overexpression mice rather than Srxn1 knockout models, the potential contributions of Srxn1 knockout to exacerbated cardiac oxidative stress and inflammation in HF remain unclear. Finally, translating findings from genetically modified mice to humans presents substantial challenges. Therefore, to substantiate our conclusions about the translational potential of HF regulators, future studies should incorporate experiments using human samples.

## 5 Conclusions

In summary, this study is the first to demonstrate the protective effects of Srxn1 against TAC‐induced cardiac oxidative stress and inflammation. Mechanistically, Srxn1 appears to inhibit TLR4/NF‐kB signalling, thereby ameliorating TAC‐induced cardiac remodelling. These findings strongly support the potential of Srxn1 as a therapeutic candidate for pathological myocardial remodelling following HF.

## Author Contributions

**Huibo Wang:** conceptualization (lead), funding acquisition (lead), resources (equal). **Ying Yang:** investigation (equal), methodology (equal), resources (equal). **Yong Ye:** data curation (equal), software (equal), validation (equal). **Xing Wei:** data curation (equal), software (equal), visualization (equal). **Shen Chen:** writing – original draft (lead). **Bin Cheng:** data curation (equal), investigation (equal), methodology (equal). **Yunbo Lv:** conceptualization (supporting), project administration (equal), writing – review and editing (lead).

## Ethics Statement

All the experimental procedures complied with the recommendations in the Guide for the Care and Use of Laboratory Animals of the National Institutes of Health (NIH Publication 8th edition, 2011) and were approved by the Ethics Committee of China Three Gorges University.

## Consent

The authors have nothing to report.

## Conflicts of Interest

The authors declare no conflicts of interest.

## Data Availability

All the data in this study are available upon reasonable request from the corresponding author.
